# Relationship between advanced maternal age and decline of endometrial receptivity: a systematic review and meta-analysis

**DOI:** 10.18632/aging.204555

**Published:** 2023-02-27

**Authors:** Jing Zhao, Bixia Huang, Ning Li, Xiaofei Wang, Bin Xu, Yanping Li

**Affiliations:** 1 Reproductive Medicine Center, Xiangya Hospital of Central South University, Changsha, Hunan, China; 2 Clinical Research Center for Women’s Reproductive Health in Hunan Province, Changsha, Hunan, China; 3 Reproductive Medicine Center, The Third Affiliated Hospital of Guangxi Medical University, Nanning, Guangxi, China; 4 Reproductive Medicine Center, Chengdu Xinan Gynecology Hospital, Chengdu, Sichuan, China

**Keywords:** advanced maternal age, endometrial receptivity, clinical pregnancy, miscarriage, oocyte donation

## Abstract

Female fertility decreases with age. A decline in oocyte quality plays a key role in reproductive problems in older women. Whether advanced maternal age (AMA) is associated with a decline in endometrial receptivity (ER) remains controversial. A systematic review and meta-analysis were conducted to evaluate the relationship between AMA and ER. Eighteen eligible studies were included in this meta-analysis. Of the 18 studies, 17, 8, 14, and 9 studies reported the impact of AMA on clinical pregnancy rate (CPR), implantation rate (IR), miscarriage rate (MR), and live birth rate (LBR), respectively. The combined results showed a trend (without significance) toward lower CPR in women with AMA than in younger women. A similar trend of worse outcomes in terms of IR was observed in women with AMA. A significantly higher MR and lower LBR were observed in infertile women with AMA than in younger women. In conclusion, there was a slightly lower IR and CPR without significance; however, significantly increased MR and decreased LBR were observed in women with AMA than in younger women, indicating that AMA is related to the decline of ER. Further prospective cohort studies with a preimplantation genetic testing for aneuploidy model are needed to observe the relationship between AMA and ER and explore the possible mechanisms.

## INTRODUCTION

With the social trend to delay childbearing because of careers, the treatment of older infertile female has become a major challenge for today’s fertility specialists. Female fertility decreases with age. A decline in oocyte quality plays a major role in reproductive problems in older women. Whether advanced age is related to the decline of endometrial receptivity (ER) remains controversial. One study reported that in women aged >40 years, both embryo viability and, to a lesser extent, ER were decreased [1].

Oocyte donation (OD) affords a scientific model to study the impact of advanced age on ER. Animal experiments have shown age-related changes in the uterus, accompanied by a decrease in implantation and pregnancy rates. Despite transferring embryos from younger animals, older animals were ultimately unable to conceive [2]. When a similar experiment was carried out in humans by OD from young healthy women to older infertile recipients, conflicting results were obtained. Some investigators have concluded that a satisfactory embryo implantation rate (IR) in women with advanced maternal age (AMA) implies that the uterine factors are not involved [3–9], while others have found decreased pregnancy rate, implantation rate, and increased miscarriage rate in recipients with advanced age, suggesting that fertility does not depend merely on oocyte age and quality, but also on uterine age [10–14].

Many clinical trials have studied the impact of AMA on the ER and results after OD. There have been no systematic reviews and meta-analyses on this issue.

Currently, there is a pressing need for a systematic review and meta-analysis to evaluate the clinical question of the relationship between AMA and the decline of ER with the OD model.

## RESULTS

### Studies selection and characteristics

**Figure 1:**
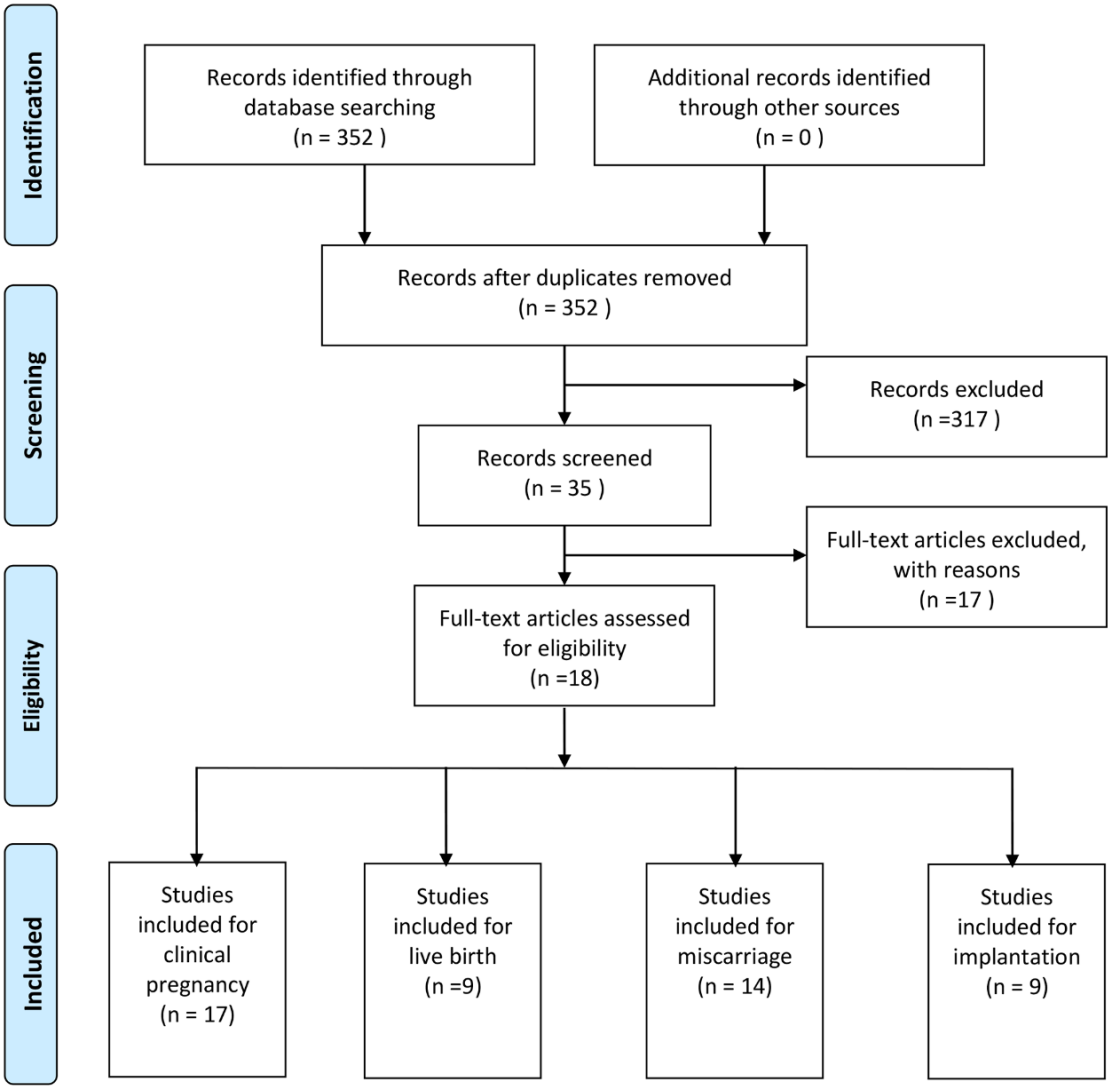
Flow chart showing study selection process.

In total, 352 articles were obtained using this research strategy. Of these, 317 articles were excluded because they were found irrelevant after reviewing their titles and abstracts. Of the remaining 35 studies, 17 were excluded for different reasons: 15 were reviews and 2 had incomplete data. Finally, 18 studies were included in the present study (Figure 1).

Eighteen eligible studies were published from 1991 to 2005, including 11 retrospective studies, 5 prospective studies, and 2 studies without a study design. The sample size ranged from 22 to 3089. Of the 18 studies, 17, 8, 14, and 9^th^ studies reported the impact of AMA on clinical pregnancy rate (CPR), IR, miscarriage rate (MR), and live birth rate (LBR) (Table 1).

**Table 1:** Characteristics of included studies.

**Study**	**Country**	**Design**	**Model**	**Protocol for COS**	**Protocol for ET**	**Sample size(cycles)**	**Group**	**Age for AMA**	**Outcomes**
Soares 2005	Spain	Retro	OD	A long protocol	HRT with or without GnRH-a	3089	<40y; 40-44y; 45-49y; >49y	45	CPR, IR, MR, obstetric outcomes
Moomjy 1999	USA	Retro	OD	GnRH-a long protocol	HRT with or without GnRH-a	370	≤34y; 35-42y;≥ 43y	42	CPR, MR
Yaron 1998	Israel	Pros	OD	HMG alone or with GnRH-a	HRT	1001	<30y; 31-40y; >40y	40	CPR, MR, LBR
Abdalla 1997	UK	Retro	OD, 2 recipients from different age group shared oocytes from a donor	Intranasal Buserelin + HMG	HRT with or without GnRH-a	104	≤39y; ≥40y	40	CPR, IR, LBR
Borini 1996	Italy	Retro	OD, Recipients of different ages shared oocytes from single donor	Buserelin/LA+FSH/HMG	HRT	114	≤39y; 40-49y	40	CPR, IR, MR
Cano 1995	Spain	Pros	OD, Recipients of different ages shared oocytes from same cohort of follicles	Long protocol with LA+HMG/FSH	HRT	90	<40y; ≥40y	40	CPR, IR, MR
Legro 1995	USA	Retro	OD	Long protocol with LA + HMG	HRT	307	≤42y; >42y	42	Ongoing-PR, MR
Rosewaks 1995	USA	/	Younger IVF-ET donor to older recipients	/	HRT	48	/	/	Ongoing-PR, IR
Balmaceda1994	USA	Retro	Donor to recipients with POF or poor responder	Long protocol with LA + HMG	HRT	258<189 fresh and 69 frozen)	≤30y;31-35y;36-40y;41-45y; 46-53y	40y	CPR, IR, MR
Check 1994	New Jersey	Retro	Infertile women undergoing IVF shared 50% oocytes to recipients	Long protocol with LA +HMG	HRT	121	<40y; ≥40y	40y	CPR, LBR
Navot 1994	USA	Pros	Younger donor to older recipients	HMG or concomitant menotropins and a GnRH-a	HRT	89	42.7±0.3 vs. 33.4±0.7	/	CPR, IR, MR, LBR
Sauer 1994	USA	Retro	Fertile young women donate Oocyte to women with different age	COH with LA and HMG	HRT	300	<30y;30-39y;40-49y.50-59y	40	IR, LBR
Abdalla 1993	UK	Retro	Oocyte donation	Intra-nasal buserelin and HMG	HRT with or without GnRH-a	371	25-29y;30-34y;35-39y;40-44y;45-49y	40	CPR, MR
Flamigni 1993	Italy	Retro	Oocyte donation	COH with buserelin/LA+FSH/HMG	HRT	141	21-35y; 36-40y; 41-49y; 50-61y	40y	CPR, IR, MR
Meldrum 1993	USA	/	Oocyte donation	COH with LA+HMG	HRT with or without LA	52	<40y; ≥40y	40y	CPR
Yaron 1993	Israel	Retro	Oocyte donation	HMG	HRT	458	<40y; ≥40y	40y	CPR, MR
Navot 1991	USA	Pros	Oocyte donation	HMG or concomitant menotropins and GnRH-a	HRT	89	<35y; >40y	40y	CPR, LBR
Sauer 1991	USA	Pros	Oocyte donation	COH with leuprolide and HMG	HRT	22	<40y;40-44y	40y	CPR, IR, LBR

### Meta-analysis

**Figure 2:**
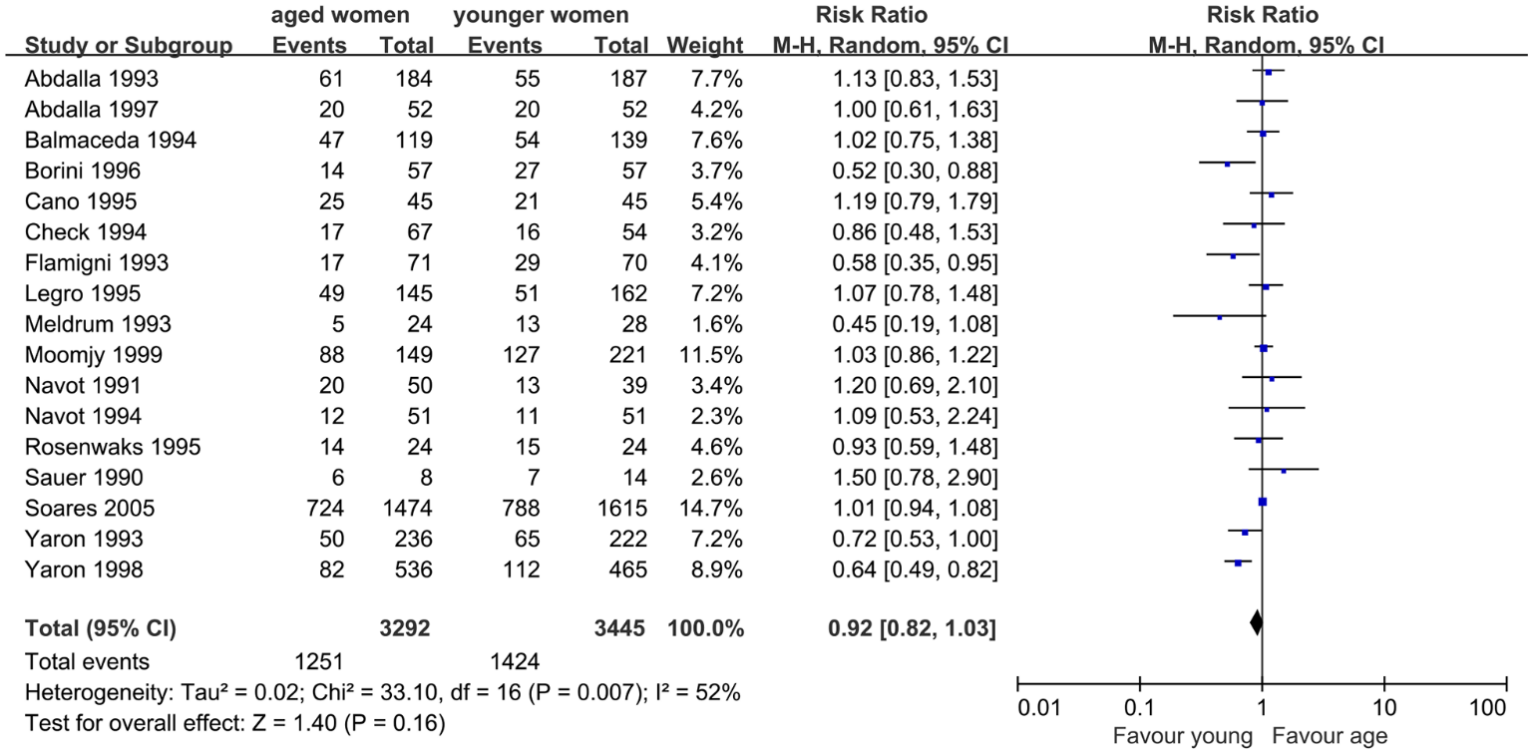
Forest plot showing the results of meta-analysis of studies comparing the effect of AMA on clinical pregnancy rate after OD treatment.

First, we evaluated the impact of AMA on CPR in infertile women undergoing assisted reproductive technology treatment using the OD model. Seventeen studies were included in this meta-analysis. The results showed a slightly lower CPR in women with AMA than in younger women (RR, 0.92; 95% CI, 0.82, 1.03; *P*=0.16). I^2^, which was used to describe the heterogeneity of the included studies, was 52%, indicating statistical heterogeneity in the results (*P*=0.007). Therefore, the random-effects model was used (Figure 2).

**Figure 3:**
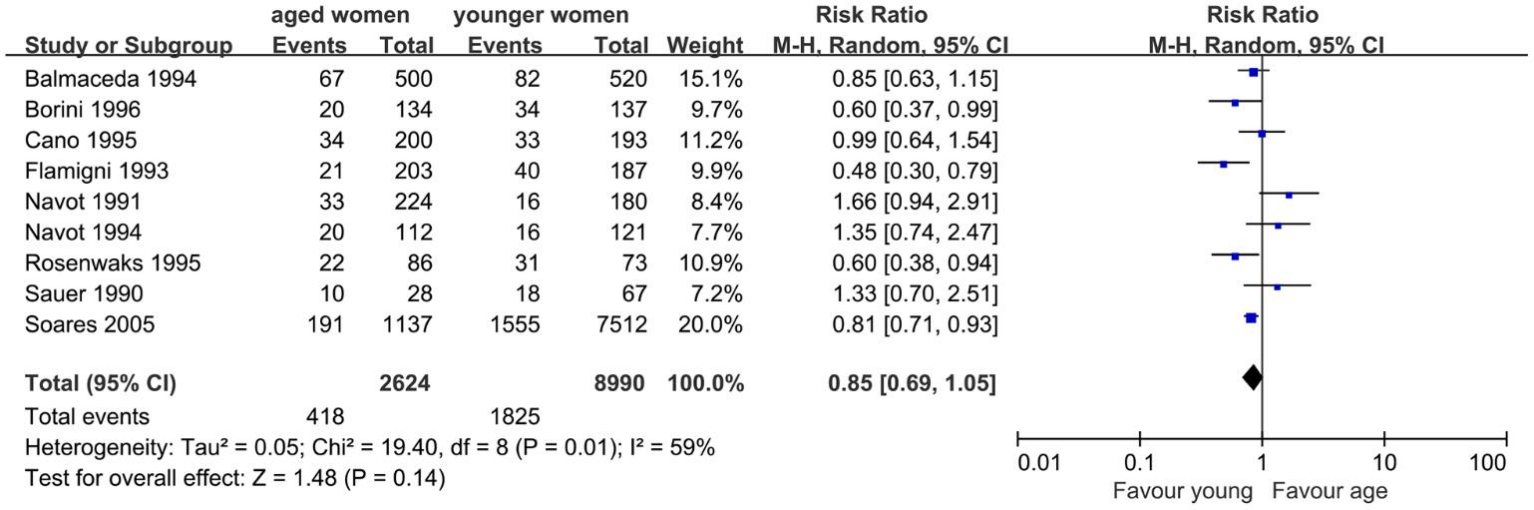
Forest plot showing the results of meta-analysis of studies comparing the effect of AMA on embryo implantation rate after OD treatment.

Similarly, nine studies were included to assess the impact of AMA on embryo implantation. The results of the meta-analysis showed similar IR in women with AMA and younger women (RR, 0.85; 95% CI, 0.69, 1.05; *P*=0.14). I^2^ was 59%, indicating moderate heterogeneity (*P*=0.01), and a random-effects model was used (Figure 3).

**Figure 4:**
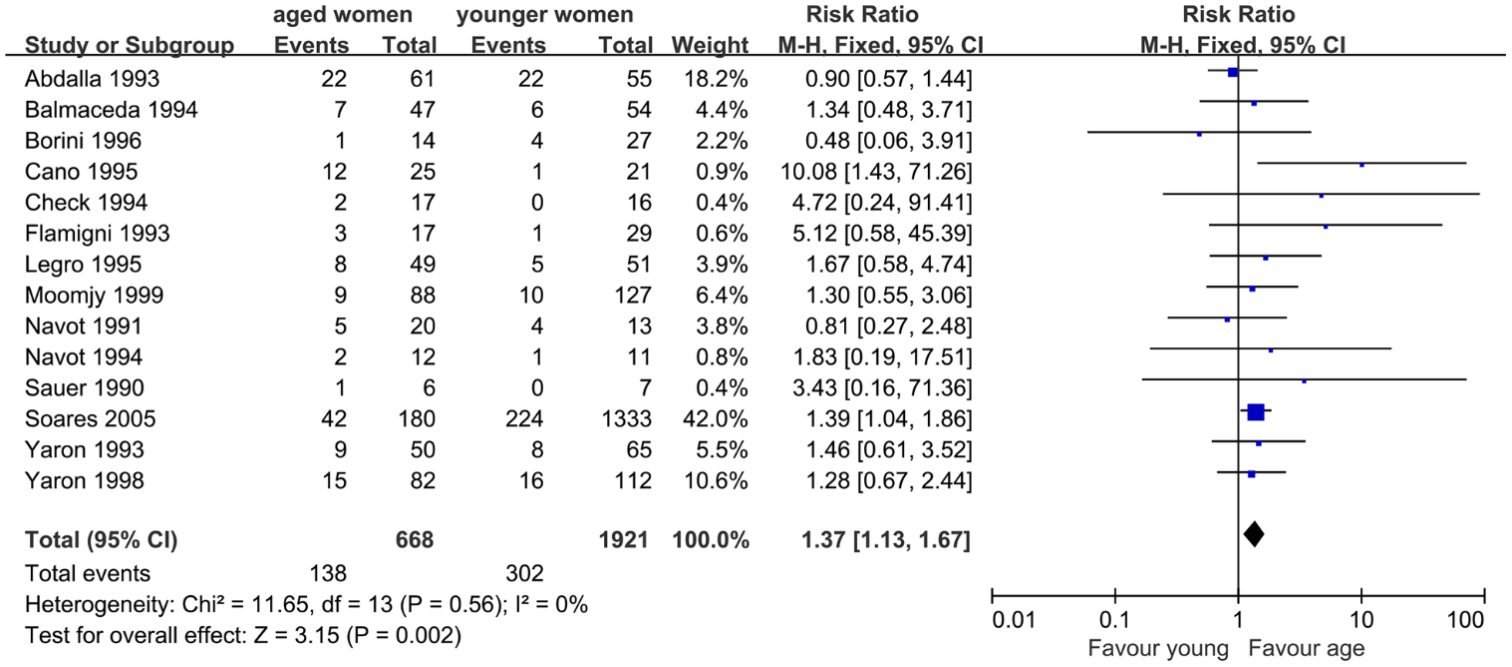
Forest plot showing the results of meta-analysis of studies comparing the effect of AMA on miscarriage rate after OD treatment.

When we evaluated the impact of AMA on MR, 14 studies were included. The results indicated a significantly higher MR in infertile women with AMA than in younger women. The Q statistic *P* > 0.1, indicated the homogeneity of the studies (I^2^=0%, *P*=0.56). The fixed effects model was used and the pooled RR was 1.37 (95% CI, 1.13, 1.67; *P*=0.002) (Figure 4).

**Figure 5:**
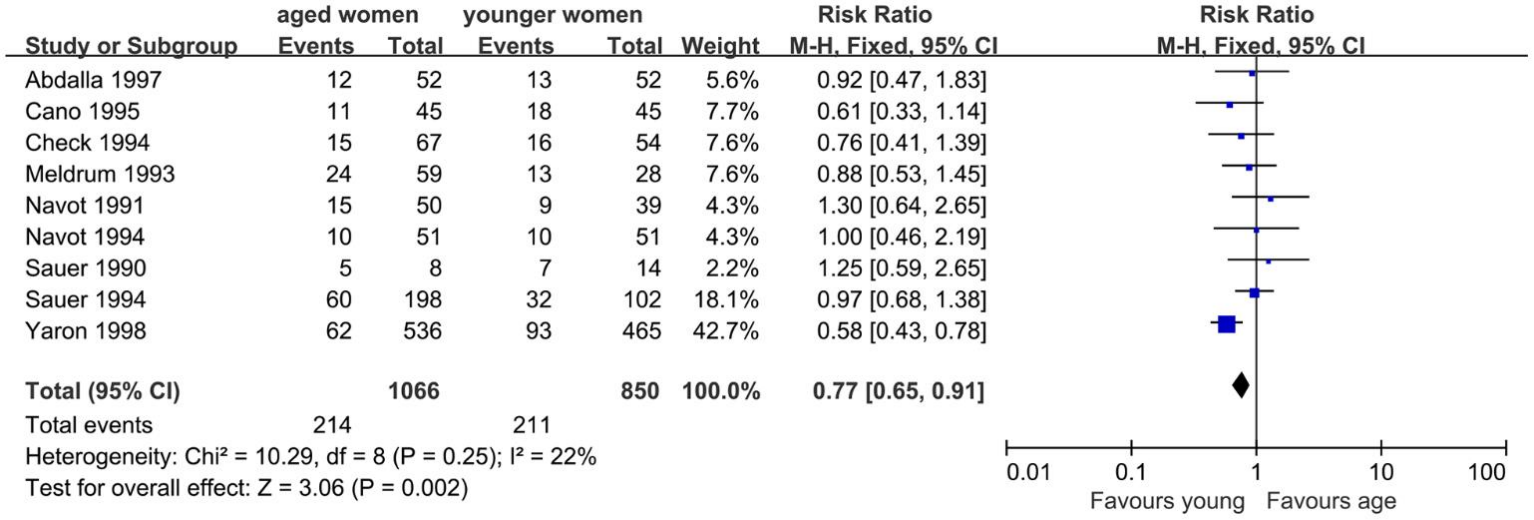
Forest plot showing the results of meta-analysis of studies comparing the effect of AMA on live birth rate after OD treatment.

Additionally, LBR was evaluated, and nine studies were included. The results of meta-analysis showed no significant difference in LBR between women with AMA and younger women. Good homogeneity was observed in the results (I^2^=22%, *P*=0.25). The fixed effect model combined RR was 0.77 (95% CI, 0.65, 0.91, *P*=0.002) (Figure 5).

The results included in this meta-analysis scored medium to high on the Newcastle-Ottawa Scale (not shown). The funnel plots evaluating the impact of AMA on CPR, IR, MR, and LBR suggest a lack of publication bias due to their symmetrical shape (Supplementary Figures 1–4).

To the best of our knowledge, this study is the first systematic review and meta-analysis to assess the impact of AMA on ER using an OD model. The results showed a trend toward a lower IR and CPR without significance; however, significantly increased MR and decreased LBR were observed in women with AMA, indicating that ER was negatively affected by advanced maternal age.

It is well known that fecundity declines in women with AMA are associated with decreased oocyte quality. However, there is no consistent conclusion regarding whether ER is also impaired in women with AMA. OD is considered a perfect model for ascertaining the extent of this relationship. Some studies have evaluated the impact of AMA on endometrial tissues using an OD model.

Early in 1990, one study explored whether ER decreased in older women. In this study, women aged 40-44 years with ovarian failure were enrolled and underwent embryo transfer with OD. These results suggest that the endometrium retains the ability to respond to gonadal steroids and receptivity for embryo implantation, even in older women [6]. Other similar studies also suggested that the age-related decline in female fertility has been attributed to oocyte quality and can be corrected by OD [4], showed similar PR, cumulative PR, and LBR in different age groups, and failed to detect any impact of age on pregnancy outcome in the OD model [15–18].

Other studies have compared pregnancy and implantation rates in oocyte recipients of different ages and showed significant differences in pregnancy and implantation rates according to the age of recipients, suggesting that the ER decreased with age [9, 10, 13]. Some studies reported significantly decreased PR [19] and IR [20, 21], significantly increased MR [11], and worse obstetric outcome [8] in women of advanced age. These discrepancies may be attributed to differences in patients’ age, body mass index (BMI), country, indication of OD, analysis method, and study design.

Although there were discrepancies among the studies, the pooled results suggest that AMA may have a negative effect on the ER. As donated oocytes are obtained from young women, the age-related decline in LBR and increased MR with OD cannot be attributed to oocyte quality. Possible explanations for our findings are as follows.

First, there is an age-related decline in ER. An *in vitro* experiment found that the expression of HOXA10, a marker of ER, was inversely correlated with uterine age [22]. An animal study compared mRNA levels of endometrial cells *in vitro* obtained from young and aged cows using next generation sequencing (NGS) and polymerase chain reaction (PCR), and found that endometrial cells of aged cows have higher levels of inflammatory, IFN-signaling, and cell division dysfunction than those of young cows [23]. In human, it has also been reported that placenta in women with AMA is associated with premature senescence during placentation due to SIRT1 deficiency, which promotes epithelial-mesenchymal transition of trophoblast cells and enhances the invasion of trophoblast cells by regulating vimentin acetylation [24]. Older women with decreased serum anti-mullerian hormone (AMH) and antral follicle count (AFC) levels showed significantly lower endometrial vascularization index, flow index, and vascularization flow index, and lower CPR and ongoing PR, indicating impaired ER [25].

Second, another possible explanation for our results may be embryo quality. The oocytes were donated by younger women, which did not contribute to poor embryo quality. However, advanced paternal age may be a reason for poor embryo quality. New dominant mutations, which may be embryologically fatal, are now known to be common in men of advanced age. Thus, it is reasonable to assume that the male partners of older recipients are more likely to be older, and such new dominant mutation may lead to decreased embryo quality [19].

Third, the risk of adverse pregnancy outcomes, such as gestational diabetes, preeclampsia, stillbirth, intrauterine growth restriction, and placenta previa, markedly increased among women with AMA. The increased complications may be related to impaired placentation function and progressive uterine vascular endothelial damage with aging [26–28].

The strength of this study is that it is the first systematic review and meta-analysis to describe the relationship between AMA and ER decline. The sample size was very large (7037 women), which provided an excellent precision for estimates with pooled RRs.

This study had several limitations. First, there was significant between-study heterogeneity, such as different study designs (prospective or retrospective studies), varied definition for AMA (40 years, 42 years, or 45 years), and different endometrium preparation protocol. In addition, most of the studies were retrospective in design, and there were residual confounding factors. Finally, some of the included studies had a small sample size. Despite these drawbacks, this systematic review and meta-analysis provide a valuable analysis and summary of the relevant literatures.

In conclusion, this study found that AMA is related to a decline in ER. Because of the small sample size and the possibility of aneuploidy embryos, further prospective cohort studies using the preimplanation genetic testing-Aneuploid (PGT-A) model are needed to observe the impact of AMA and analyze the possible causes.

## MATERIALS AND METHODS

This study was conducted in accordance with the Preferred Reporting Items for Systematic Reviews and Meta-Analyses guidelines. As the data were extracted from previously published studies, and our paper did not include animal and human studies, institutional review board approval was an exemption.

### Search strategy

A comprehensive search of PubMed, EMBASE, and Google Scholar was conducted from their inception dates until May 2022. The keywords used for the search were as follows: a term including advanced age (advanced maternal age, older woman, aging woman) and a term that included the endometrial receptivity (uterine receptivity). These subsets were summarized with “AND” to obtain the most complete literatures related to our study object. Cohort, retrospective, and prospective studies published in English were included. Full-text review and data extraction were completed by two separate reviewers, and any disagreements were resolved by consensus or by a third reviewer.

### Study selection and data extraction

After reviewing the retrieved titles and abstracts, irrelevant studies were assessed using the Newcastle-Ottawa Quality Assessment Scale. Data were extracted by two authors independently using pre-defined criteria. Data extraction included the research features and results.

### Statistical analysis

Review Manager Version 5.3 was used for meta-analysis. Categorical variables were calculated using the Mantel-Haenszel statistical method and expressed as risk ratio (RR); Forest plots were used to assess the heterogeneity of the included studies, and I^2^ was used to quantify the heterogeneity between studies. A fixed or random-effect model was used to calculate RR and its 95% confidence interval (CI). Because of the low power of the χ^2^ test for heterogeneity with a small sample size, a *P* value of 0.10 rather than 0.05 was considered to be statistically significant.

## Supplementary Material

Supplementary Figures
