# Supplementary material for: Relationship between advanced maternal age and decline of endometrial receptivity: a systematic review and meta-analysis
Source: Aging (Albany NY). 2023 Feb 27;15(7):2460–72. doi: 10.18632/aging.204555 (PMC10120912; doi:10.18632/aging.204555)
Supplement: Supplementary Figures [file aging-15-204555-s001.pdf]

SUPPLEMENTARY FIGURES

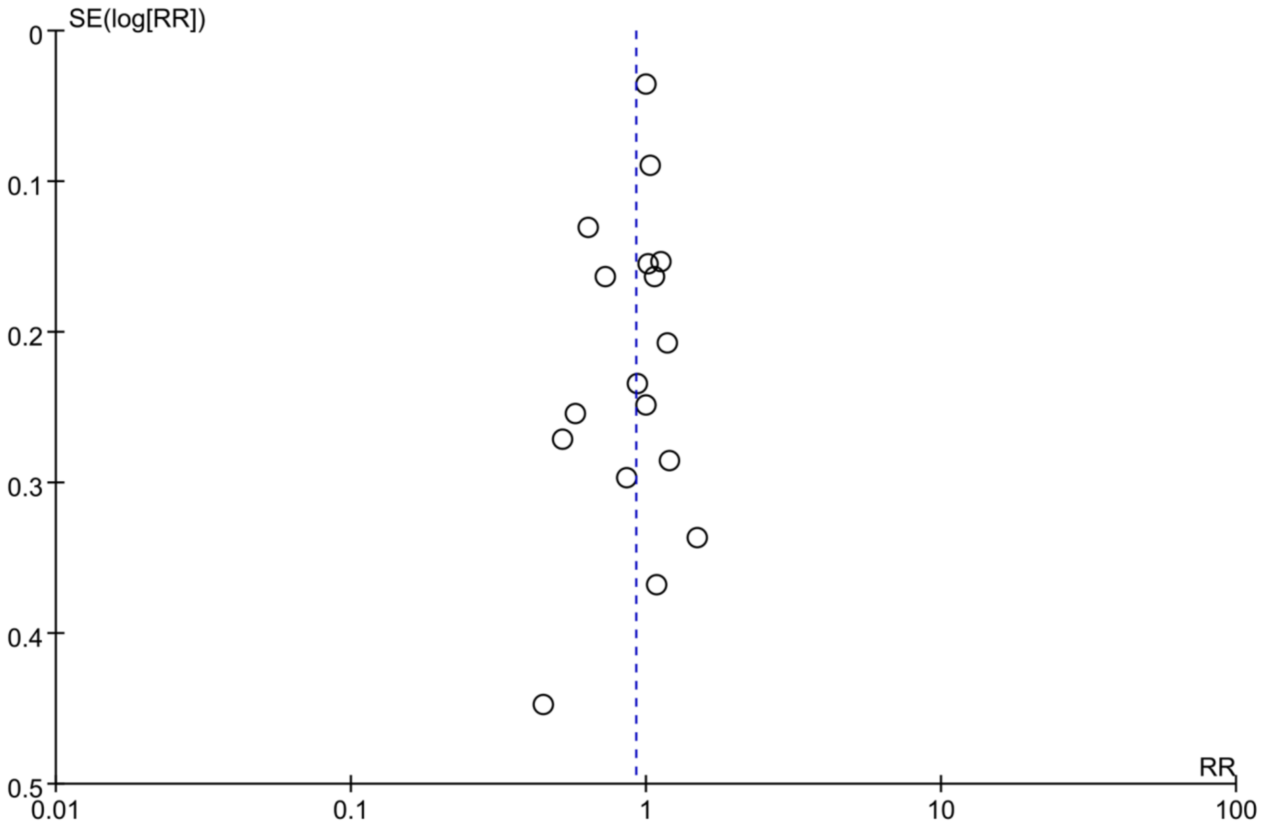

Supplementary Figure 1. Funnel plot of analysis for the effect of AMA on clinical pregnancy rate after OD treatment.

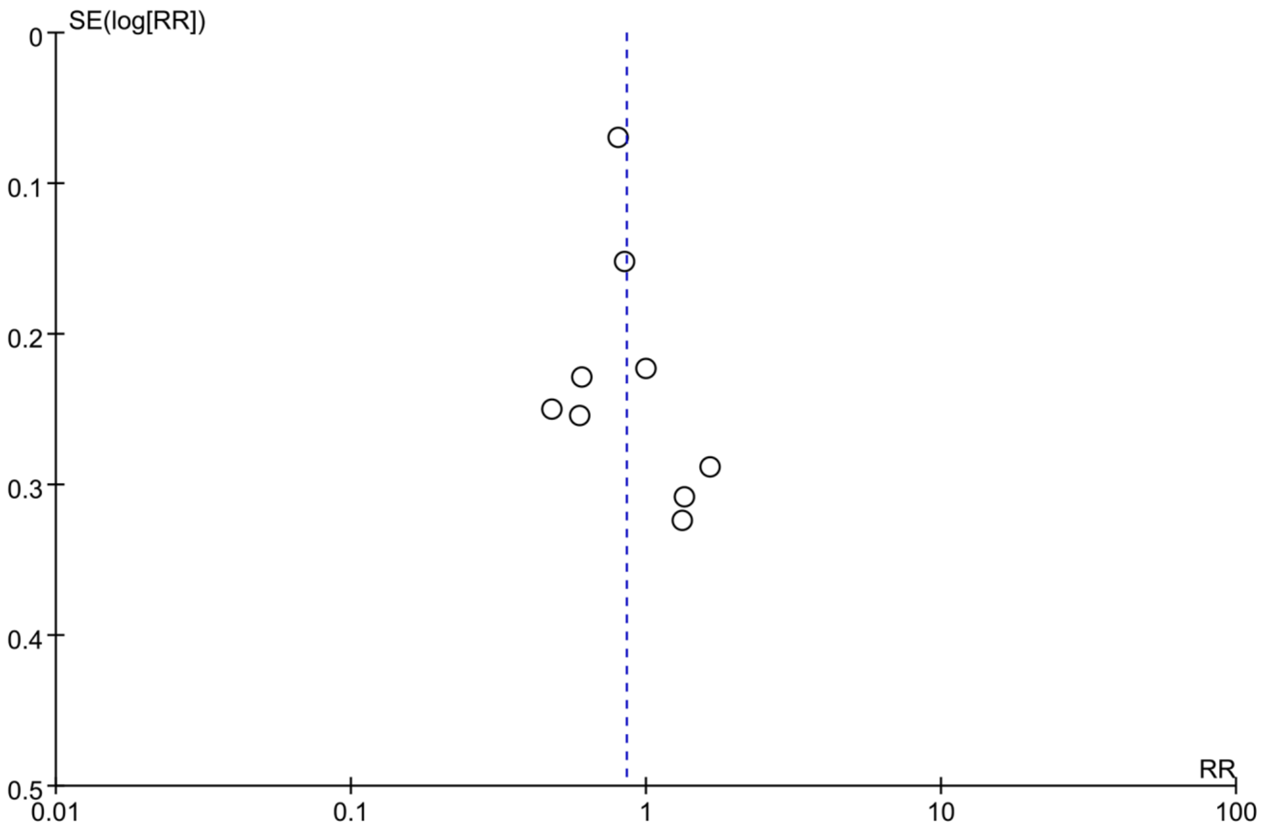

Supplementary Figure 2. Funnel plot of analysis for the effect of AMA on embryo implantation rate after OD treatment.

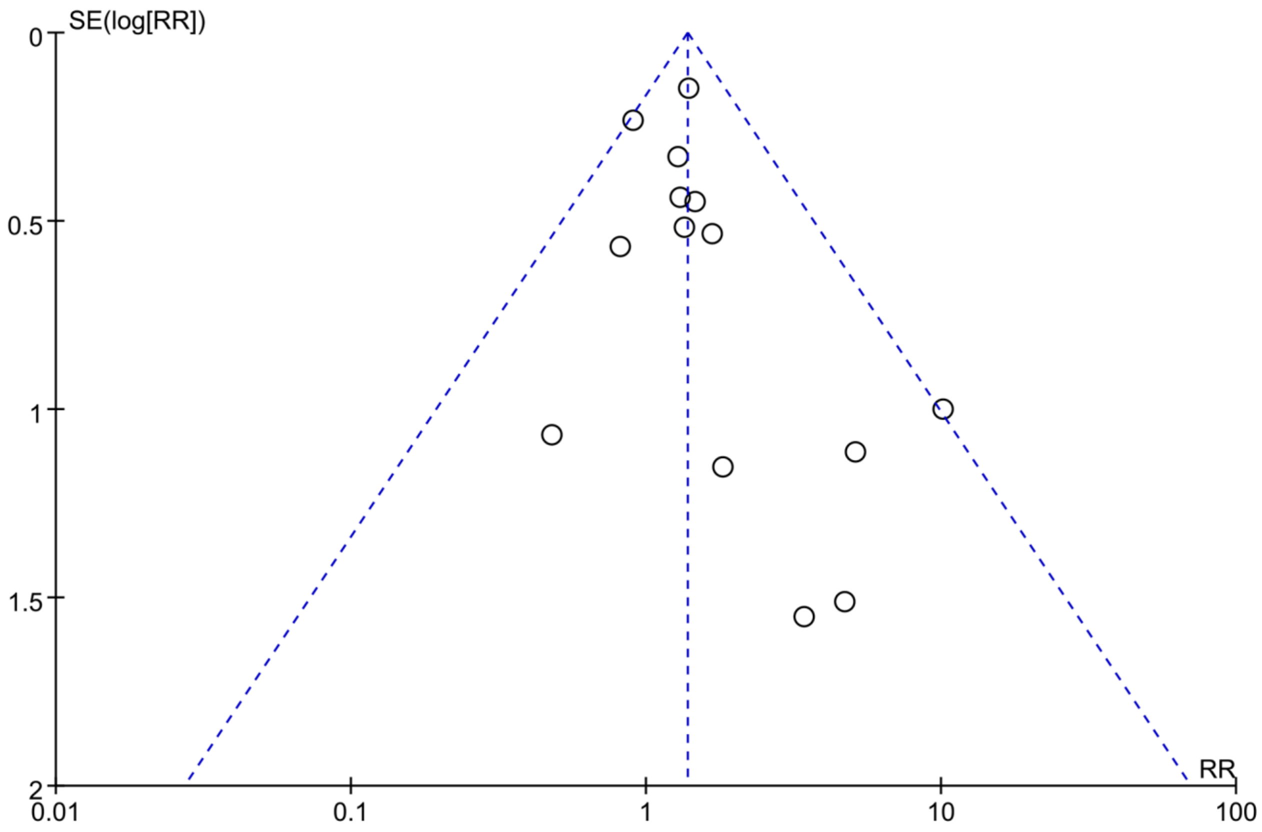

Supplementary Figure 3. Funnel plot of analysis for the effect of AMA on miscarriage rate after OD treatment.

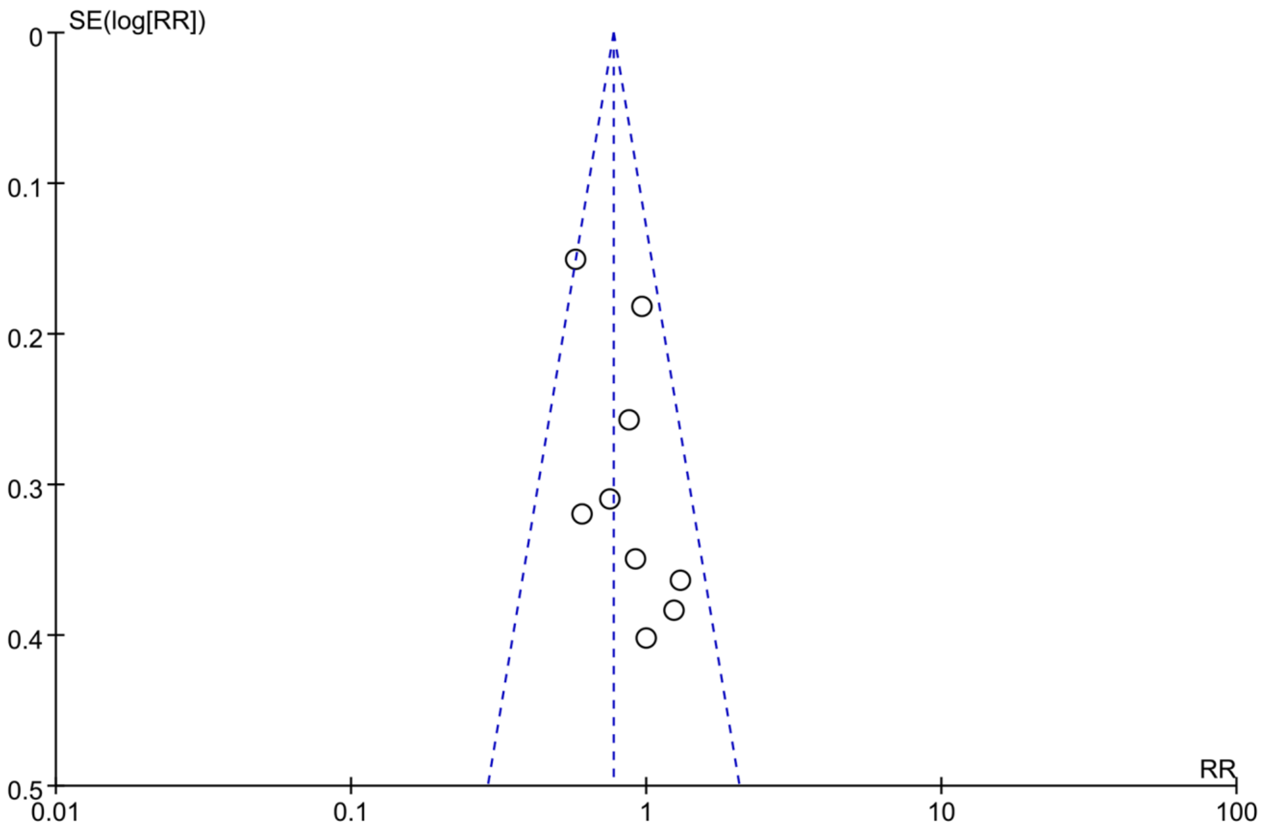

Supplementary Figure 4. Funnel plot of analysis for the effect of AMA on live birth rate after OD treatment.
